# Magnetic Resonance Imaging for the diagnosis and management of acute colonic diverticulitis: a review of current and future use

**DOI:** 10.1002/jmrs.458

**Published:** 2021-02-19

**Authors:** Franziska Jerjen, Tooba Zaidi, Shannon Chan, Ajay Sharma, Reuel Mudliar, Khadija Soomro, Yobelli Jimenez, Warren Reed

**Affiliations:** ^1^ Medical Imaging Optimisation and Perception Group (MIOPeG) Discipline of Medical Imaging Science Sydney School of Health Sciences Faculty of Medicine and Health Franziska Jerjen and Tooba Zaidi are joint first authors The University of Sydney Sydney NSW Australia

**Keywords:** Colon, computed tomography, diagnosis, diverticular disease, magnetic resonance imaging

## Abstract

Diverticular disease is one of the most common causes of outpatient visits and hospitalisations across Australia, North America and Europe. According to the Gastroenterological Society of Australia (GESA, 2010), approximately 33% of Australians over 45 years of age and 66% over 85 years of age have some form of colonic diverticulosis. Patients with colonic diverticulosis are known to develop subsequent complications such as acute colonic diverticulitis (ACD), and when more than one attack of diverticulitis occurs, there is a 70‐90% chance that the individual will experience ongoing problems and recurring infections throughout their lifetime. Medical imaging is fundamental in the diagnosis, treatment and ongoing management of ACD and its complications, with Computed Tomography (CT) identified as the prevailing gold standard in the last few decades. Cross‐database searching highlighted a large gap in the literature regarding the effectiveness of Magnetic Resonance Imaging (MRI) as a non‐ionising radiation alternative imaging tool for ACD imaging after the mid‐2000s, despite ongoing technological advancements in this modality. This narrative review identified 13 key publications (11 primary prospective cohort studies, 1 systematic review and 1 meta‐analysis) that evaluate MRI for ACD imaging, of which five were published within the last decade. Several existing MRI protocols are deemed suitable for ACD imaging, and it is recommended they be re‐evaluated in larger cohorts. Future studies should consider the rapidly growing technological improvements of MRI, its cost efficiency and its applicability in modern day healthcare settings when addressing ACD management. This is especially important considering the gradual rise in radiation dose among the Australian population attributable to increased CT referrals, alongside increased reporting of ACD cases in younger individuals.

## Graphical abstract

Diverticular disease is one of the most common causes of outpatient visits and hospitalisations. Cross‐database searching has highlighted a large gap in the literature: an absence of publications providing efficacy data on Magnetic Resonance Imaging (MRI) as a non‐ionising radiation alternative for ACD. This narrative review aims to explore available published data and provide an up‐to‐date evaluation of MRI as a tool for the diagnosis and ongoing management of ACD.

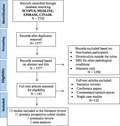

## Introduction

Diverticular disease is an umbrella term for a spectrum of conditions associated with diverticulosis which are small, pressure‐induced herniations (diverticula) at points of weakness within the mucosal and submucosal lining of the digestive system.^1^ There is a general trend of higher prevalence of diverticulosis in Australia, the United States of America, Canada and parts of Europe compared to Asian and African countries.^1^ This disparity between countries suggests that environmental factors play a significant role in this disease. Fibre deficiency, seen more commonly in industrialised countries,^1^ has been implicated as the primary determinant for colonic diverticulosis. Other possible explanations have been discussed in greater depth previously, including age‐related mucosal wall thickening, intraluminal pressures, colonic structural abnormalities, genetic predisposition, prolonged gastrointestinal transit time, obesity and lack of physical activity.^2^, ^3^

Diverticulosis of the colon is traditionally described as an age‐related disease, seen in approximately 33% of Australians over 45 years of age and in 66% of Australians over 85 years of age.^4^ Patients with colonic diverticulosis are known to develop subsequent complications such as acute colonic diverticulitis (ACD).^4^, ^5^ More than one occurrence of ACD has been associated with a 70‐90% risk of ongoing problems and recurring episodes of infection throughout an individual’s lifetime.^4^ Given the recurring nature of ACD in patients with a history of diverticulitis, it is concerning that recent global reports suggest increased incidences of ACD among younger individuals.^6^, ^7^, ^8^

Diagnosis of colonic diverticular disease and ACD requires a combined assessment of clinical signs, biomarkers and imaging studies. The current preferred imaging modality is Computed Tomography (CT). CT enables accurate assessment of intraluminal and extraluminal components of the disease and the involvement of nearby organs. However, in the last two decades, concern has grown over increased radiation dose attributable to CT, along with the associated increased risk of cancer.^9^, ^10^, ^11^

Magnetic Resonance Imaging (MRI) is a non‐ionising imaging alternative to CT. During the late 20th and early 21st century, many publications assessed MRI use for ACD. However, following the establishment of CT as the gold standard imaging modality for lower abdominal pain,^12^, ^13^, ^14^, ^15^, ^16^ few subsequent publications in the last 10 years have reassessed MRI’s evolving and future potential in great depth. The lack of high‐powered randomised and blinded studies providing supportive data for MRI in ACD imaging is reflected in current clinical guidelines, wherein CT is favoured over this modality.^17^, ^18^

Increased cases of ACD worldwide, particularly in younger individuals, combined with the need for repeat imaging of recurrent infection events, suggest a need to re‐evaluate the appropriateness of MRI versus CT. In light of continual and rapid technological advancements and patient needs, this narrative review aims to explore available published data and provide an up‐to‐date evaluation of MRI as a tool for the diagnosis and ongoing management of ACD. Patient comfort, financial expenses and the overall burden of MRI on medical imaging departments will also be considered when defining its future feasibility.

## Methods

A narrative literature review was deemed most appropriate to provide a broad perspective of the existent knowledge on MRI use in the diagnosis and management of ACD, given the lack of data available to inform a detailed meta‐analysis. A literature search was conducted in four databases, Scopus, Embase, MEDLINE and CINAHL, using boolean combinations of keywords such as ‘MRI’, ‘magnetic resonance imaging’, ‘diverticul*’, ‘colon*’, ‘large intestine’ and ‘large bowel’. The inclusion criteria were studies published in the English language, focusing on human adult participants of all genders and ages, with or without pre‐existing conditions. Studies focusing on diverticular disease outside the large bowel were excluded, as were paediatric studies, which predominantly focused on Meckel’s diverticula of the small bowel. The search was limited to publications within the last 20 years (2000–2020).

## Selection Process

**Figure 1:**
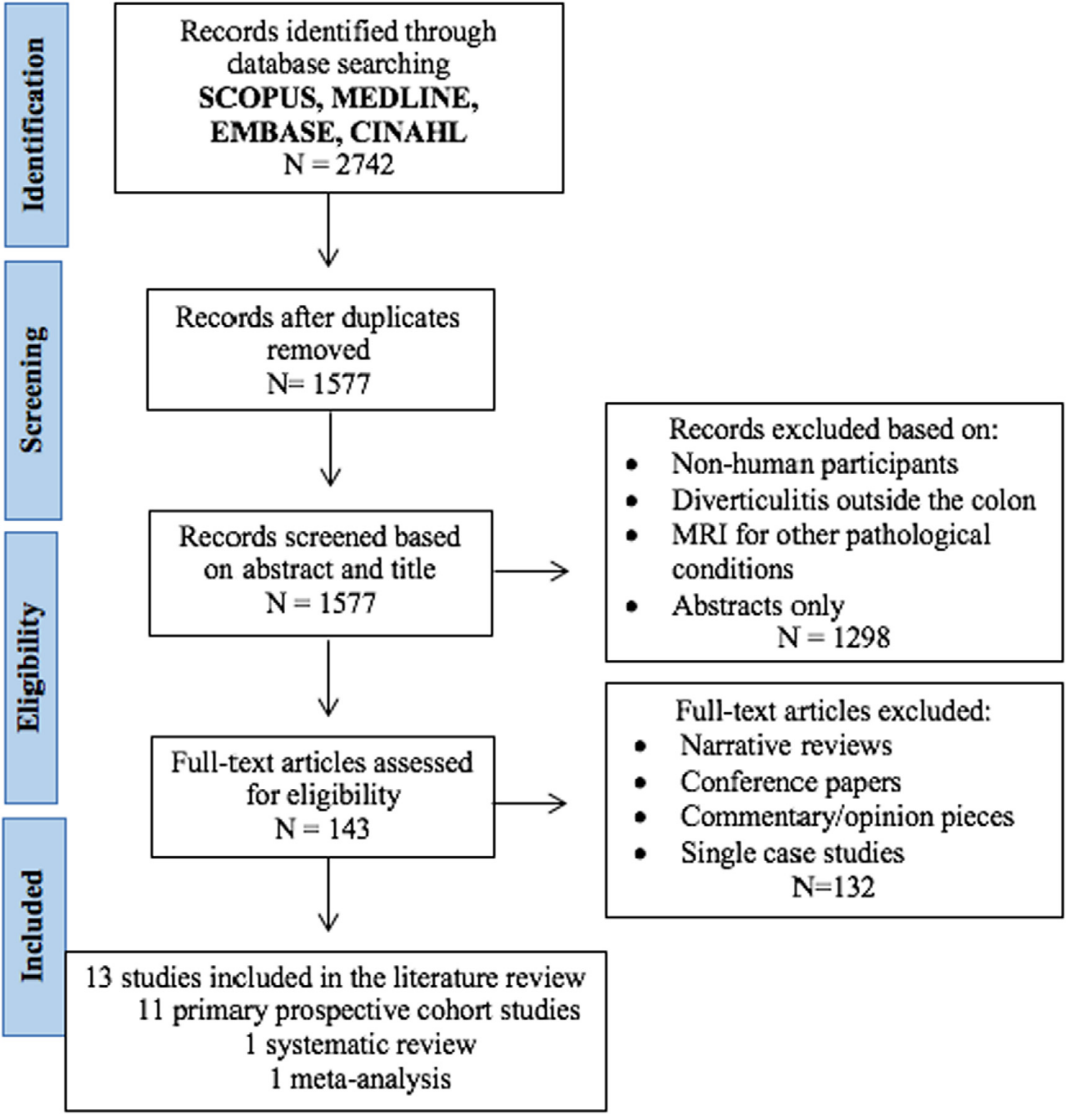
Flow Diagram for literature selection process.

The Preferred Reporting Items for Systematic Reviews and Meta‐Analyses (PRISMA) was appropriated to inform the selection process of literature (Fig. 1). Upon removal of duplicates, the remaining 1577 papers’ titles and abstracts were reviewed by six authors (FJ, TZ, SC, AS, RM, KS). Narrative review articles, conference papers, commentary or opinion pieces were excluded. Studies that compared CT against a reference standard on consecutive or randomly selected patients were sought, thus excluding single‐case studies. A total of 13 studies, 11 primary prospective cohort studies, one systematic review and one meta‐analysis were identified (Table 1). Data extracted from sourced studies included sample size, study classification, methodology and key findings.

**Table 1:** Summary of published studies investigating MRI for ACD.

Author (ref)	Study Design	Sample size (*n*), age range, gender distribution	Sensitivity Specificity (%)	Diagnostic reference Standard	Key findings
Ajaj et al.^5^	Prospective cohort	*n* = 40, 55‐77 years of age	Sensitivity: 86% Specificity: 92%	CC with biopsy	Moderate evidence for diagnostic utility of gadolinium‐enhanced MRI with excellent internal validity. High external validity for sigmoid diverticulitis.
Andeweg et al.^19^	Systematic Review and Meta‐analysis	N/A	Not reported.	N/A	^25^ reported by the authors in their systematic review
Byott and Harris.^20^	Prospective cohort	*n* = 468 (*n* = 13 for diverticulitis)	Not specified.	US	Exploratory study with good reference standards.
Cobben et al.^21^	Prospective cohort	*n* = 5, 28‐83 years of age, M/F: 2/3	Not specified.	US, CT	Preliminary evidence for differential diagnosis of right‐sided colonic diverticulitis against appendicitis using non‐contrast MRI.
Halpenny et al., 2009 ^22^	Prospective cohort	*n* = 26, 42‐74 years of age, M/F: 11/15	Sensitivity: 100% Specificity: 100%	CT	Confirms the findings of Heverhagen et al., 2008 with high internal and external validity demonstrated.
Heverhagen et al.^23^	Prospective non‐randomised cohort	*n* = 23, 42‐75 years of age, M/F: 10/13	Not specified.	US	Feasibility study with detailed non‐contrast MRI protocol for initial investigation of ACD. Strong participant retention and follow‐up. MRI demonstrated to be tolerable and a fast scan time of under 5 minutes is of highlight.
Heverhagen et al.^24^	Prospective cohort	*n* = 20, 40‐69 years of age	Not specified.	US with CT or; Endoluminal US; or Surgery	Replication of previous findings on T2 MR protocols provides moderate evidence for MRI’s diagnostic utility. Small non‐randomised sample with low external validity.
Heverhagen et al.^25^	Prospective cohort	*n* = 55, 29‐76 years of age, M/F: 29/26	Sensitivity: 94‐96% Specificity: 88%	Surgery and pathology and/or CT	High intra‐ and inter‐rater reliability demonstrated for diagnosis of ACD by MRI. Robust methodological evidence for MRI as a diagnostic tool.
Liljegren et al.^26^	Systematic Review	N/A	Re‐calculated sensitivity: 83% and specificity: 81% from Ajaj et al ^5^	N/A	One study (Ajaj et al ^5^) met the inclusion criteria of assessing MRI against a diagnostic reference modality in randomised consecutive patients and was categorised to have good reference standards (CEBM Level 2b) permitting re‐calculation of reported sensitivity and specificity values. Studies that did not report sufficient data for re‐calculation were excluded.
Oiastamo et al.^27^	Randomised cohort study	(*n* = 30); *n* = 15 suspected sigmoid diverticulitis, 39‐75 years of age, M/F: 9/6 and; *n* = 15 sigmoid cancer, 57‐82 years of age, M/F: 6/9	Sensitivity: 100% Specificity: 100%	CT	Demonstrated differential and superior capability of MRI to diagnose sigmoid diverticulitis against sigmoid cancer in comparison to CT. Non‐contrast DWI MR protocol highlighted. Confidence intervals and efficacy measures not detailed.
Romagnoli^28^	Randomised cohort study	(*n* = 16); *n* = 8, uncomplicated diverticular disease, 50‐73 years of age, M/F: 4/4 and; *n* = 8, healthy controls, 44‐67 years of age, M/F: 6/2	N/A	CC for diverticular disease group	Efficacy study with good reference standards for evaluating the role of MR‐defecography in patients with diverticular disease.
Schreyer et al.^12^	Prospective cohort	*n* = 14, 42‐74 years of age, M/F: 9/5	Not specified.	CT	Demonstrated identical identification of diverticulosis and diverticulitis on MRI in comparison to CT. 3D reconstruction via virtual colonoscopy demonstrated with low external validity due to pre‐existing confounding risk factors in patients. Low internal validity due to lack of blinded evaluation of MRI images.
Storz et al.^29^	Prospective inception cohort	*n* = 393, 46‐65 years of age, M/F: 226/167	Not specified.	Not performed	Population‐specific study of risk factors not diagnostic accuracy associated with ACD. Excellent intra‐ and inter‐rater reliability demonstrated for MRI in a preventative setting for a population without MRI contraindications. Demonstrated extent of diverticular disease to be associated with age.

*ACD: Acute colonic diverticulitis, CC: conventional colonoscopy, CEBM: The Oxford Centre for Evidence‐Based Medicine levels of evidence, CT: computed tomography; DWI: Diffusion‐Weighted Imaging, MRI: Magnetic resonance imaging, US: Ultrasound*.

## Discussion

### Diagnostic accuracy of MRI in ACD imaging

One of the salient advantages of MRI over CT and ultrasound (US) is its superior soft tissue resolution,^12^, ^24^, ^27^ which allows for accurate identification of pathological changes in ACD. These include the presence of pericolonic fat stranding, bowel wall thickening and inflammation, mesenteric infiltration, stenosing of colonic segments and diverticula growth. Some studies argue that findings gleaned from MRI and CT are extremely similar, particularly the demonstration of diverticula, abscesses and the formation of fistulas, thus making it a viable non‐ionising alternative to CT.^5^, ^12^, ^13^ Previous studies have reported sensitivity and specificity values of MRI in the diagnosis of ACD ranging between 86‐96% and 88‐92%, respectively.^5^, ^25^ However, the actual ranges may be lower than reported, as found by Liljergen et al.,^26^ who re‐calculated the sensitivity and specificity findings reported by Ajaj et al.,^5^ (Table 1). No explanation was provided by the original authors for this discrepancy. Two studies have achieved a sensitivity and specificity of 100% in smaller homogenous cohorts.^22^, ^27^ However, due to limitations in methodological design (small sample size and use of poor/non‐independent reference standards) across the studies identified by this review, the empirical data remain insufficient to alter current guidelines.^18^ Of note, Andeweg and colleagues^19^ categorised the Heverhagen et al.^25^ study to be of moderate quality in their systematic review, but were unable to conduct a meta‐analysis for the diagnostic accuracy of MRI due to a lack of comparable studies. This is further reflected in the Appropriateness Criteria (ACR) recommendations where MRI is identified as *‘Might be Appropriate’* as an imaging procedure for initial examination of left lower quadrant pain and suspected diverticulitis.^17^

**Figure 2:**
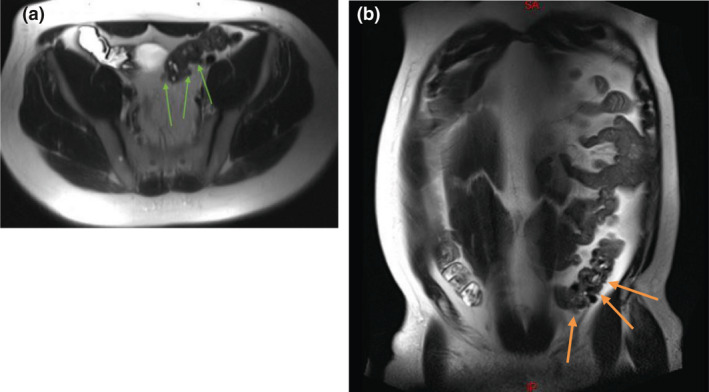
**(**A) Axial and (B) coronal images using T2 Half Fourier Acquisition Single Shot Turbo Spin Echo (HASTE) sequencing demonstrate characteristic diverticular outpouchings (arrows) of the sigmoid colon (permission obtained to reproduce images).

Five studies published in the last decade build upon existing data and, in particular, highlight the improvements in non‐contrast MRI techniques for detecting ACD pathology.^12^, ^20^, ^27^ For example, Byott and Harris^20^ demonstrated the reliability of T2 Half Fourier Acquisition Single Shot Turbo Spin Echo (HASTE) sequences in acute abdominal imaging, including a subset of patients with colonic diverticulitis. This rapidly acquired MRI sequence can reduce motion artifacts, addressing the susceptibility of MRI to free air and motion and permitting demonstration of the characteristic diverticular outpouching with superior soft tissue differentiation (Fig. 2). Figures 2A and 2B show characteristic signs of ACD (diverticular outpouching, bowel wall thickening and fat stranding), achieved using an abdominal T2 HASTE sequence in axial and coronal planes during an MRI scan.

Although the approach tested by Heverhagen and colleagues^23^ results in longer acquisition times, they report greater sensitivity to inflammatory changes and ascites (hallmark characteristics of ACD) with the use of T2‐fat suppression sequences. Whilst Byott and Harris’^20^ findings were extrapolated from populations of adequate size (total of 468 cases and 116 correctly diagnosed cases), Heverhagen et al.’s^23^ findings clearly demonstrate inherent bias due to a lack of reasonable study size (10 of 11 patients correctly diagnosed). Future studies encompassing larger cohort sizes are needed to demonstrate MRI’s potential suitability in diagnosing colonic diverticular disease. Pending said studies, these preliminary findings may be extrapolated to predict that non‐contrast MRI sequences are superior in the differentiation of colonic diverticular disease from colorectal cancers when compared to CT and US.^12^, ^27^, ^30^ Furthermore, Storz et al.^29^ reported high inter‐ and intra‐rater reliability for detecting diverticular disease and discerning possible pathological mechanisms in all colonic segments in its earliest stages. In light of the primary papers identified herein, the developments in MRI for ACD imaging can be broadly discussed with respect to contrast‐enhanced and non‐contrast‐enhanced MRI protocols, as follows.

### Contrast‐enhanced MRI

Two of the primary papers identified in this literature review, Heverhagen et al.^25^ and Ajaj et al.^5^, demonstrated that MRI coupled with intravenous gadolinium has a high sensitivity and specificity in the diagnosis of ACD as detailed in Table 1.^5^, ^25^, ^31^As a contrast agent, gadolinium displays an initial vascular phase and is inclined to migrate into the interstitium, allowing observation of colonic wall thickening (determined by wall thickness exceeding 3 mm on the short axis of the large bowel lumen) and the presence of pericolic fat stranding. As such, it is highly useful when detecting other abdominal pathologies such as neoplasm, appendicitis, epiploic appendicitis, ischaemic colitis and other chronic inflammatory bowel diseases.^14^ Most importantly, contrast enhancement allows the differentiation of colonic diverticulitis from its main differential diagnosis: primary colon carcinoma.^19^ However, Ajaj et al.^5^ also demonstrated MRI colonography's lack of sensitivity in differentiating between invasive carcinomas smaller than 5 mm and inflammatory lesions associated with sigmoid diverticulitis, despite the use of gadolinium. Eight false‐positive cases were reported in this study^5^. The study further identifies limitations of conventional colonoscopy, as the alternative modality to be used for patients with complicated diverticulitis: the risk of perforation and the possibility of being inconclusive due to heavy interstitial stenoses. Future studies are needed to build on the sensitivity and specificity data for differentiating small invasive carcinomas against inflammatory lesions of ACD. This will inform the suitability of contrast‐enhanced MRI for patients with complicated diverticulitis who are at risk of colon cancer.

As with all contrast‐enhanced MRI, gadolinium use can be contraindicated in patients presenting with acute renal failure, chronic kidney disease or an estimated glomerular filtration rate below 30 ml/minute/1.73 m^2^.^32^ Recent clinical studies have further cited the risk of toxicity from deposition of linear gadolinium‐based contrast agents (GBCA) in patients with normal renal function. Whilst GBCA deposition within brain, liver, skin and bones have been noted, no robust evidence correlates any adverse physiological effects with said deposition. Although the effects of GBCA deposition remain unclear, their use should still be critically evaluated based on long‐term risks and benefits.^33^, ^34^, ^35^, ^36^, ^37^

### Non‐contrast MRI

Multiple studies show promising results from non‐contrast MRI protocols for initial investigation of suspected ACD in patients presenting with lower left quadrant pain.^21^, ^23^, ^24^ Cobben et al.^21^ identified characteristic outpouchings of the right‐sided colon with adjacent colonic wall thickening and surrounding fat stranding in a prospective cohort of 5 patients following clinical suspicion of appendicitis. The feasibility of rapid acquisition sequences was further detailed by Heverhagen et al.^23^ for suspected sigmoid diverticulitis, as characteristic signs of ACD were observed in all 11 patients included in their study. More recently, Oistamo et al.^27^ reported 100% sensitivity and specificity in differentiating sigmoid diverticulitis against sigmoid carcinoma, by combining T2‐weighted and diffusion‐weighted imaging (DWI) sequences. In comparison, a sensitivity of 67% and specificity of 93% were reported using CT. Although all studies were underpowered due to small sample sizes, these non‐contrast MRI techniques provide valuable insight for developing evidence‐based MRI protocols for the differential diagnosis of ACD.

While an in‐depth analysis of MRI protocols is beyond the scope of this review, the value of DWI sequences in the imaging of ACD deserves mention. Formerly used for oncological procedures and highlighting neoplastic processes, DWI protocols create contrast dependant on the Brownian movement of water molecules within tissue. The produced signals of tissues exhibiting cellular swelling/restricted diffusion are depicted as hyperintense signals on scans. DWI allows for improved detection of oedema, abscess formation and inflammatory lesions, which suggests a potentially high sensitivity for colonic diverticulitis.^27^, ^38^ Nonetheless, addressing DWI’s longer image acquisition time and susceptibility to motion artefacts is necessary before its implementation in clinical practice for ACD.

### Developments in MRI technique

In the last few decades, numerous techniques have evolved to overcome the long‐standing challenges posed by intraluminal gas and peristaltic motion of abdominal structures in MRI imaging. Smooth muscle relaxants such as Butylscopolaminiumbromid (Buscopan) have been administered in a number of studies (Table 1) at a dosage of 40 mg intravenously to control for peristaltic bowel motion and colonic spasms prior to the acquisition of non‐enhanced T1‐weighted sequences.^5^, ^12^, ^23^, ^24^, ^25^ Due to the contraindications for Buscopan in patients with pre‐existing conditions such as glaucoma, glucagon hydrochloride may alternatively be administered prior to intravenous contrast to reduce discomfort and bowel peristalsis.^5^ Ajaj et al.^5^ have further recommended the use of anticholinergic drugs specifically to improve the effectiveness of post‐processing techniques such as digital subtraction on colonographic MRI data sets.

Additionally, Byott and Harris^20^ offer a non‐invasive, three‐pronged solution by combining the rapidly acquired T2 HASTE to reduce motion artefacts with a T2 fat‐saturation sequence to increase sensitivity in identifying inflammatory changes, as well as a gradient echo sequence for the assessment of free gas. Future comparison with CT acquisition would be useful to shape standard practices involving MRI. The development of rapid single shot MRI sequences, such as the T2 HASTE, and the continual advancements in DWI scanning have significantly reduced scan times.^20^, ^39^ MRI sequences that previously required 20 minutes of table acquisition time have almost halved, and in some cases are comparable in duration to that of an abdominal CT scan.^12^, ^20^, ^23^

A recent review by Serai et al.^40^ further highlights five newly developed and promising MRI techniques capable of reducing physiologic motion in abdominal imaging. These include compressed sensing, simultaneous multi‐slice excitation, radial imaging, and motion‐resolved imaging in combination with non‐cartesian sampling and compressed sensing reconstruction. Techniques such as these have decreased the sensitivity of MRI to artefacts produced by free air and motion and can be implemented by future studies.

## MRI in Clinical Practice

Despite improvements in technology and efforts to address the challenges of MRI, limitations persist which prevent its use from superseding CT as the gold standard for ACD imaging.^5^, ^25^, ^27^. Such prevailing challenges include the following: contraindications in patients with MRI unsafe implants, claustrophobia, higher initial and ongoing maintenance costs of equipment, as well as limited availability relative to CT and US.

### Patient care

The radiographer first and foremost has a duty of care towards the patient during image acquisition. A positive patient experience depends heavily on appropriate management of the patient’s emotional and physical states. Proper patient communication and preparation are particularly important for MRI scanning to ensure patient compliance and mutual understanding of the imaging procedure. Advancements in rapid acquisition single shot sequences coupled with post‐processing algorithms have demonstrated shortened scan times and address limitations of claustrophobia and motion artefacts, thereby improving patient experience and image quality.^20^, ^39^, ^41^

### Financial issues

Despite MRI advancements to improve patient comfort and satisfaction, a prevailing impediment to its wider use in medicine and patient access is the relatively large out‐of‐pocket expense compared to that of CT and US. In Australia, numerous MRI examinations are excluded from the Medicare Benefits Schedule^42^ Whilst the out‐of‐pocket expense for an MRI scan has seen a sharp increase from A$143 to A$184 between the 2010‐11 to 2015‐16 period as reported by the Australian Diagnostic Imaging Association, the average expense for other imaging procedures was estimated to be roughly A$97.11 during the 2016‐17 period.^42^

### Additional limitations

The inability of MRI to facilitate interventional techniques (biopsy and drainage) is considered to be another significant drawback. Current literature and clinical practice predominantly recommend two methods for the treatment of diverticular abscesses: antibiotic therapy, prescribed for phlegmon/abscesses less than 4 cm in diameter, or treatment by drainage if larger than 4 cm, performed under US or CT guidance.^43^ The ability of CT and US to support these interventional techniques is a significant advantage over MRI.^32^, ^44^, ^45^ However, the successful practice of MRI‐guided biopsy and percutaneous procedures has been demonstrated in other areas, predominantly breast and neuromuscular imaging.^46^, ^47^ It would therefore be prudent to re‐evaluate the role of MRI in ACD and assess the potential to translate these techniques to abdominal/pelvic imaging.

The implementation of MRI also burdens imaging departments with the need for further training of radiographers in this modality. This is due to the additional technological expertise required to perform MRI examinations. MRI radiographers must be able to interpret pathology and competently manipulate scan variables, which depend on both physical and biochemical parameters to differentiate between soft tissue pathologies.^48^ This requirement for expertise ultimately decreases patient turnover rates in some imaging departments, as patients may have to wait until a qualified radiographer is available to perform the procedure. However, this may encourage the advancement of the diagnostic radiography profession by encouraging radiographers towards training in an additional modality.

## Summary and Future Directions

The clinical efficacy and diagnostic accuracy of MRI in the context of ACD remain unclear. The reasons are three‐fold – firstly, current evidence supporting the relationship between the severity of ACD and the diagnostic accuracy of MRI protocols is scarce. Secondly, pitfalls in the current literature on MRI are largely attributed to small prognostic inception cohorts at early stages of the disease and lack longitudinal data from follow‐up imaging. Thirdly, there is an absence of cross‐population representation with studies displaying low external validity due to the use of small homogenous inception cohorts, with the majority of data acquired from patients with sigmoid diverticulitis. Findings from the published studies included in this review provide low to moderate evidence for MRI as a diagnostic tool in favour of CT and US for ACD imaging.^17^, ^19^ It would be beneficial to further investigate the use of MRI, CT and US across large randomised prospective trials to establish an optimal imaging pathway for diagnosing ACD. Due to MRI’s superior capability in identifying earlier stages of diverticular disease and lack of ionising radiation, researchers are further encouraged to implement fast sequence MRI techniques alongside CT and US to build on the scant data available. The use of a single standardised diagnostic test across studies is also encouraged to establish consistency.

Future investigations should focus on looking at the economic feasibility of MRI in clinical practice. It would be advantageous to have professional bodies such as The Royal Australian and New Zealand College of Radiologists (RANZCR) investigate MRI use in ACD for potential establishment of protocols and associated healthcare expenses. Ultimately, the potential of MRI in future clinical practice should be empirically driven. This potential will ultimately be determined by two key issues: firstly, the ability to reduce image acquisition time to improve motion‐related artefacts, by taking advantage of the intrinsic contrast properties of the underlying pathological changes to minimise the need for gadolinium enhancement; secondly, the success of implementing protocols that focus on improving costs and service availability to patients through the Medicare Benefits Scheme.

## Conclusion

This narrative review investigated the current and potentially growing role of MRI in the diagnosis and ongoing management of patients with ACD. MRI offers consistent imaging performance, with the added benefits of modern cross‐sectional imaging without the burden of ionising radiation. This review highlights the unavailability of research into MRI for ACD imaging, with only a few clinical papers produced in the early 2000s, and even fewer after 2010. While it appears that CT prevails as the modality of choice for ACD, the current review highlights the potential of recently developed MRI techniques as alternatives. Based on the literature analysis, the authors encourage the scientific community to continue exploring the potential of MRI for ACD management and as a diagnostic tool in populations affected by ACD, especially younger patients. This will support evidence‐based practice and encourage practitioners to take advantage of opportunities where non‐ionising imaging techniques can be used. Randomised prospective trials evaluating MRI against CT as a diagnostic standard would be particularly helpful in guiding the appropriate choice of imaging for evaluating ACD in future.

## Conflict of Interest

The authors declare no conflict of interests regarding this literature review.
